# Relations between childhood psychological maltreatment and mental health dimensions within a higher-order model

**DOI:** 10.1016/j.ijchp.2023.100416

**Published:** 2023-10-07

**Authors:** Zhuoni Xiao, Ingrid Obsuth, Franziska Meinck, Aja Louise Murray

**Affiliations:** a Department of Psychology, University of Edinburgh, Edinburgh, UK; b Clinical & Health Psychology, University of Edinburgh, Edinburgh, UK; c School of Social and Political Science, University of Edinburgh, Edinburgh, UK; d Faculty of Health Sciences, North-West University, Vanderbijlpark, South Africa; e School of Public Health, University of the Witwatersrand, Johannesburg, South Africa

**Keywords:** Psychological Maltreatment, Psychological abuse, Psychological Neglect, Psychopathology, Trans-diagnostic psychopathology

## Abstract

**Background:**

Experiences of childhood psychological maltreatment have been found to be associated with various mental health outcomes, and this association persists into adulthood.

**Objective:**

This study investigated whether some types of psychological maltreatment are more harmful than others; whether the harms associated with different types of psychological maltreatment are generalized or specific to particular domains of psychopathology; and whether the associations vary by gender.

**Method:**

Participants (*N* = 544, 63.9 % mother as primary caregiver) were Chinese adults from various regions in China. Participants completed measures of childhood psychological maltreatment experiences perpetrated by their primary caregiver and the mental health outcomes of depression, anxiety, anger, physical aggression, and hostility. The data were analyzed in a hierarchical model in which depression and anxiety were defined as indicators of an internalizing factor, while anger, physical aggression, and hostility were defined as indicators of an externalizing factor. Internalizing and externalizing then defined a higher-order general psychopathology factor. The results suggested equivalent harms of psychological abuse and psychological neglect. Further, the associations between psychological maltreatment and mental health were not unique to specific symptom domains but showed broadband associations with general psychopathology.

**Results:**

These findings suggest that trans-diagnostic interventions may be the most effective approach for addressing the mental health impacts of psychological maltreatment.

**Conclusion:**

Childhood psychological maltreatment may pose a broadband risk for any and all forms of psychopathology.

## Introduction

Childhood psychological maltreatment is common and can have negative impacts on adult mental health. Indeed, a recent systematic review (Xiao et al., 2022a) reported associations between different forms of psychological maltreatment (i.e., emotional abuse and emotional neglect) and a range of adult mental health outcomes such as depressive disorder (Neumann, 2017), anxiety symptoms (Sunley et al., 2020), low self-esteem (Arslan, 2016) and borderline personality disorder (Fung, Chung & Ross, 2020) in adults.

According to Vachon et al. (2015), there are four assumptions made in relation to child maltreatment: (1) harmfulness (i.e., that child maltreatment causes substantial harm), (2) non-equivalence (i.e., that the harmfulness of child maltreatment varies by type of maltreatment), (3) specificity (i.e., that specific forms of child maltreatment have specific consequences), and (4) non-universality (i.e., that there are gender and race differences among the effects of child maltreatment). They tested each of these and found evidence only for the harmfulness assumption for all non-sexual child based maltreatment types, and not equivalence, non-specificity, and universality. However, research on these assumptions for different forms of psychological maltreatment (i.e., psychological abuse, psychological neglect, and psychological non-support) needs to be explored. The current study aims to explore assumptions (2) non-equivalence, (3) specificity, and (4) non-universality using a large sample of Chinese adults who reported on their childhood psychological maltreatment experiences and current mental health.

Building on previous evidence that has confirmed the harmfulness of childhood psychological maltreatment, an important question is whether different forms of psychological maltreatment are equally harmful (the ‘non-equivalence assumption’). A key distinction within psychological maltreatment is between psychological abuse, psychological neglect, and a lack of psychological support. The past evidence for the non-equivalence assumptions in the context of childhood psychological maltreatment is generally supportive of the assumption. For instance, Muniz et al. (2019) found that childhood psychological abuse, not psychological neglect was highly correlated with externalizing but not internalizing problems. Further, Vahl et al. (2016) found that childhood psychological neglect was less strongly related to internalizing problems than childhood psychological abuse. Compared with psychological abuse and psychological neglect; however, psychological non-support has received less attention (Briere, Godbout, & Runtz, 2012). Psychological non-support refers to a caregiver failing to provide gestures or acts of caring, acceptance, and assistance towards a child (Shaw et al., 2004). Previous studies have shown that less psychological support by caregivers is linked to several forms of psychopathology (Xiao et al., 2022b). Conversely, psychological support is associated with fewer posttraumatic stress disorder symptoms after experiencing events such as natural disasters (Bokszczanin, 2008). However, the links between psychological non-support and mental health have not yet been fully explored. Therefore, it is important to investigate its associations alongside those of more widely researched forms of psychological maltreatment, namely psychological abuse and psychological neglect.

Whether the specificity assumption holds in the context of childhood psychological maltreatment also remains unclear. In the extant literature, there is some evidence that psychological maltreatment confers broadband risks for mental health. For instance, Vahl et al. (2016) found childhood psychological abuse and psychological neglect were associated with internalizing and externalizing problems, rather than with specific domains of mental health. In addition, Heleniak et al. (2016) found similar associations between internalizing and externalizing problems and psychological abuse. However, a recent study found evidence that psychological abuse was more likely related to externalizing than internalizing problems (Muniz et al., 2019). Therefore, the extent to which associations between psychological maltreatment and mental health outcomes are generalized or disorder-specific is still unclear. This is complicated by the fact that different mental health domains tend to be correlated with one another (Murray, Eisner & Ribeaud, 2016). As such, research that disentangles the unique versus generalized associations between psychological maltreatment and mental health outcomes is particularly needed to provide illumination on this issue.

In regard to the non-universality assumption, a key dimension on which there may be differential effects of psychological maltreatment on mental health outcomes is gender. Vahl et al. (2016) found positive associations between psychological neglect and internalizing problems in boys but only non-significant associations in girls. However, Hagbrog, Tidefors and Fahlke (2017) did not find gender differences in the associations between psychological maltreatment and externalizing problems. As such, there also remains a need for further research to clarify the nature and extent of any gender differences in the impact of psychological maltreatment on mental health outcomes.

Recently, mental health research has been moving towards trans-diagnostic perspectives that acknowledge that there is considerable co-occurrence between common mental health and behavioral issues in both children and adults (Forbes et al., 2020). These perspectives have highlighted the dimensions of internalizing and externalizing problems as broadband factors that can capture the co-occurrence between a range of different symptoms. The internalizing dimension captures the co-occurrence between disorders such as depression and anxiety, while the externalizing dimension captures the co-occurrence of issues such as aggression and conduct problems. The general factor of psychopathology is a further extension that captures the co-occurrence of internalizing and externalizing problems (Forbes et al., 2020). It has been suggested that the general factor of psychopathology can be explained by the influence of non-specific causal factors that increase risk of all symptom domains of psychopathology, both internalizing and externalizing (Lahey et al., 2017).

Several studies have explored the associations between environmental stressors and psychopathology using a hierarchical model of psychopathology similar to the above-described (Forbes et al., 2016; Kotov et al., 2017). This includes a number of studies using this framework to examine the impacts of childhood adversity on psychopathology. For instance, Meyers et al. (2015) found that early exposure to traumatic events was related to broader internalizing and externalizing problems. Keyes et al. (2012) found that the effects of child maltreatment on common psychiatric disorder were fully mediated through the latent liability dimensions, with an impact on underlying liability levels to internalizing and externalizing level, rather than specific psychiatric disorders. A more recent study (Conway et al., 2018) found nearly equivalent prospective effects of early family stress on overarching internalizing and externalizing dimension, but no evidence of disorder-specific effects. Taken together, early childhood adversity experiences had non-specific associations with psychopathology, increasing the risk for symptoms of various mental disorders via their associations with broad trans-diagnostic factors, but did not appear to have unique relations with specific symptom domains (Conway et al., 2019).

Building on these findings and taking a hierarchical approach to modeling mental health would allow us to examine the specificity of the associations between forms of psychological maltreatment and specific mental health symptoms domains as well as the broader relationships with higher order trans-diagnostic factors. In addition, it would allow us to compare the strength of these associations, as well as test whether gender could be moderators among these associations. Furthermore, we used the Chinese community samples in the current study, as there are a lack of studies outside Western context and it is important to gather more globally representative data on the potential impacts of psychological maltreatment.

## Current study

In the current study, we aimed to address the questions: (1) whether the impacts of different types of childhood psychological maltreatment are the same on domains of psychopathology – *Q1: (non-) equivalence of harm*? (2) whether these associations are unique to specific domains of psychopathology (i.e., depression, anxiety, anger, physical aggression, and hostility), or act at the level of broader domains (i.e., internalizing or externalizing behaviors), up to the most generalized broadband risks for psychopathology – *Q2: outcome specificity*? (3) does gender moderate the strength of the associations between psychological maltreatment and psychopathology - *Q3: gender invariance and gender differences*?

Thus, the primary objective of this study was to address these gaps in the literature using a hierarchical model in a Chinese adult sample. To the authors’ knowledge, this is the first study that explored the associations between different domains of mental health in adults and their relations with childhood psychological abuse, psychological neglect, and psychological non-support.

Hypotheses were based on the literature mentioned above, it was expected that psychological abuse and psychological neglect would be more strongly associated with psychopathology than psychological non-support. It was also expected that different forms of psychological maltreatment would have broadband associations with general psychopathology and only small or non-significant unique associations with specific domains of psychopathology (i.e., depression, anxiety, anger, physical aggression, and hostility). Finally, gender invariance was expected – in line with most of the previous literature that has found no significant differences in the associations between different types of psychological maltreatment on psychopathology (Vahl et al., 2016).

As previous research has shown that adverse childhood experiences such as physical abuse, sexual abuse or household dysfunction can have negative impacts on adult mental health outcomes (see Hughes et al., 2017 for review), there were adjusted for in our models.

## Method

### Ethics

This research was approved by the Ethics Committee (317–1902/8). Participants were provided with an information sheet before taking part and gave informed consent before participating.

### Sample and procedures

A general community sampling approach was used in the present study. Participants were not specifically recruited based on their experiences of psychological maltreatment or mental health problems. Five hundred forty-four participants (60 % aged 21–30; 63.2 % females) were recruited via social media in China and completed an online questionnaire utilizing the Qualtrics platform. Participants were offered two pounds as compensation. A full description of the demographic characteristics of the participants are provided in Table S1 in Supplementary Materials.

### Types of psychological maltreatment

The Chinese version of the Psychological Maltreatment Review (PMR; Briere et al., 2012) was used to measure psychological maltreatment. This measure was translated into the Chinese language and validated in a previous study by Xiao et al. (2022b). Participants were asked to rate the frequencies of behaviors by caregivers that they experienced before the age of 18 on a response scale from 0 (never) to 6 (over 20 times per year).

*Psychological Abuse* was measured by ten items (e.g., “*Said mean thing about you*” or “*Embarrassed you in front of people*”). Cronbach's alpha in this sample was 0.90, with McDonald's omega was 0.93.

*Psychological Neglect* was measured by ten items (e.g., “*Didn't do things they said they would do for you*” or “*Acted like you weren't there, even though you were*”). Cronbach's alpha was 0.90, with McDonald's omega was 0.91.

*Psychological Non-support* was measured by eight items (e.g., “*Tried to make you feel better when you were upset or hurt*” or “*Praised you when you did something good*”). Cronbach's alpha was 0.87, with McDonald's omega was 0.91.

### Types of psychopathology symptoms domain

Five domains of psychopathology including two symptoms’ measurements and three behavioral problems were assessed. These were selected as core domains of psychopathology with a common onset in childhood and adolescence and persistence into adulthood (Lahey et al., 2004).

*Depression* was measured by the Patient Health Questionnaire-9 (PHQ-9; Kroenke, Spitzer & Williams, 2001). In the current study, we adopted the Chinese version from our previous study (Xiao et al., 2022b). Example items include “*Trouble concentrating on things, such as reading the newspaper or watching television*” or “*Thoughts that you would be better off dead or of hurting yourself in some way*”. The measure has excellent psychometric properties in English (Kroenke et al., 2001) as well as in Chinese (Wang et al., 2014). Participants were asked to indicate whether they suffered from those symptoms over the past two weeks on 0 (not at all), 1 (several days), 2 (more than half the days), and 3 (nearly every day). Cronbach's alpha was 0.87 in this sample, with McDonald's omega was 0.87.

*Anxiety* was measured by the Clinical Anxiety Scale (CAS; Snaith et al., 1982). In the current study, the Chinese version of CAS was adopted from Xiao et al. (2022b). Participants were asked to respond from 0 (rarely none of the time) to 4 (most or all of the time). Example items such as “*Due to my fears, I avoid social situations, whenever possible*,” or “*I used tranquilizers or antidepressants to cope with my anxiety*”. Previous studies have shown acceptable reliability for the scale's scores (Kim et al., 2017). Cronbach's alpha was 0.93 in this sample, and McDonald's omega was 0.95.

*Physical aggression* was measured by the Buss-Perry Aggression Questionnaire – physical aggression sub-scale (BPAQ; Buss & Perry, 1992). Nine items with a 5-point Likert-scale from 1 (extremely uncharacteristic of me) to 5 (extremely characteristic of me), were used to assess participants' physical aggression level. Example items include: “*I get into fights a little more than the average person*” and “*Given enough provocation, I may hit another person*”. Cronbach's alpha was 0.79, with McDonald's omega was 0.84 in the current sample.

*Anger* was measured by seven items used a 5-point Likert-scale from the BPAQ – anger sub-scale. Example items such as “*Sometimes I fly off handle for no good reason*” or “*I sometimes feel like a powder keg ready to explore*”. Cronbach's alpha was 0.83 and McDonald's omega was 0.87 in the current sample.

*Hostility* was measured by eight items using a 5-point Likert-scale from the BPAQ – hostility sub-scale. Example items such as “*I know that ‘friends’ talk about me behind my back*” or “*I am suspicious of overly friendly strangers*”. Cronbach's alpha was 0.86, with McDonald's omega was 0.86 and McDonald's omega was 0.89 in the current sample.

### Control variable

Adverse Childhood Experiences (ACE). Ten items using dichotomous response (i.e., yes or no) were used to assess participants’ childhood adversity which included child abuse and household dysfunction (adopted from the previous study by Xiao et al., 2022b). Example items included: “*Were your parents ever separated or divorced*” and “*Did a household member go to prison*”. In the data analysis, we removed questions that related to emotional abuse and emotional neglect to avoid overlap with the PMR, therefore, only 8 items of ACE were included in the data analysis. Cronbach's alpha for the eight items was 0.76, with McDonald's omega was 0.81 in the current sample.

### Data analysis

**Fig. 1:**
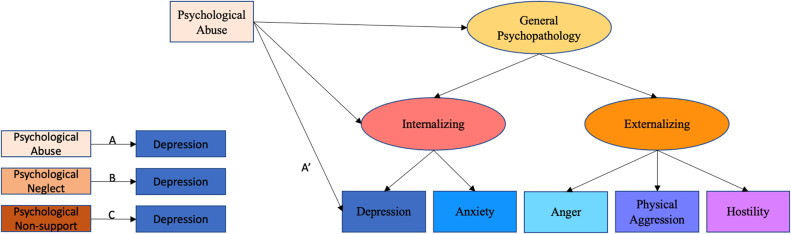
An example of the paths. Paths A, B, and C each represent total effects for a specific type of childhood psychological maltreatment with a specific symptom domain of psychopathology. Path A’ represents a direct effect, after controlling for higher-order factors (i.e., the association unique to depressive symptoms, specifically).

Descriptive analyses were conducted in SPSS. The structural validity of the psychopathology of different domains was explored by confirmatory factor analysis (CFA) using the ‘lavaan’ package in R statistical software (Rosseel, 2012). Adequate-fitting model by conventional fit criteria (CFI > 0.90, TLI >0.90, RMSEA < 0.08, RSMR < 0.09; Kline, 2015) were taken as evidence for factorial validity. The higher-order model was tested using robust maximum likelihood estimation (MLR). An internalizing factor was specified representing the common variance of depression and anxiety, and an externalizing factor was specified representing the common variance of anger, physical aggression, and hostility. A general psychopathology factor specified representing the common variance in internalizing and externalizing problems (see Fig. 1).

Analyses for (non-) equivalence of harm were based on testing for differences in the strength of the association for each type of psychological maltreatment with each domain opf psychopathology. For example, paths A, B, and C in Fig. 1 represented the total effects for the associations between experiences of psychological abuse, psychological neglect, and psychological non-support with depressive symptoms. Multiple linear regressions were conducted to test (non-) equivalence of harm.

Analyses for outcome specificity were based on examining whether these psychological maltreatment-psychopathology associations were related to specific domains of psychopathology (i.e., depression, anxiety, anger, physical aggression, and hostility), to the overlap among these symptom domains (i.e., in the boarder common factors of internalizing or externalizing), or to general psychopathology. Specifically, each total effect was compared with its corresponding direct effect. Direct effects represented the unique associations between each type of psychological maltreatment and each domain of psychopathology after controlling for the shared variance captured in higher-order factors in the model. Multiple multivariate regressions were fit within the higher-order model. For example, path A’ in Fig. 1 represented a direct effect – the part of the total effect between psychological abuse and depression that is unique to depression specifically. In contrast, the differences between path A and path A’ is the indirect effect, which refers to the part of the total effect that is accounted for by internalizing and general psychopathology (i.e., not unique to depression).

Analyses for gender invariance were explored in each model as a moderator. Specifically, Satorra-Bentler chi-squared difference testing (Satorra & Bentler, 2010) and a critical value of 0.01 change in the comparative fix index (CFI; Cheung & Rensvold, 2002) were used to compare models that allowed parameters to vary by gender and models that constrained parameters to equality by gender.

In order to control for adverse childhood experiences, the eight items ACE sum score was controlled for in the hierarchical models as other adverse childhood experiences may affect mental health (Jones et al., 2018). Code for all models is provided at: https://osf.io/h3n7k/?view_only=cf6430b215634ed3b007ef91baf2fd7f.

## Results

### Inferential statistics

Table 1 showed the descriptive statistic and correlations between variables. Table S2 in the Supplementary Material showed the ANOVA of ACE on different age group, and results suggested that there are no significant differences between different age group on ACE scores.

**Table 1:** Descriptive statistics and correlations between variables.

	Mean	SD	PA	PN	PNS	ACE	Anxiety	Anger	Physical Aggression	Hostility	Depression
PA	18.89	13.50	1	.756	.242	.347	.356	.368	.391	.347	.419
PN	15.05	12.49	<0.001	1	.410	.356	.441	.324	.366	.345	.441
PNS	18.22	11.37	<0.001	<0.001	1	.121	.306	.051	.037	.049	.127
ACE	0.90	1.69	<0.001	<0.001	.006	1	.297	.252	.237	.264	.323
Anxiety	30.72	15.21	<0.001	<0.001	.000	<0.001	1	.509	.473	.474	.696
Anger	18.56	5.12	<0.001	<0.001	.261	<0.001	<0.001	1	.694	.620	.444
Physical Aggression	22.89	5.71	<0.001	<0.001	.401	<0.001	<0.001	<0.001	1	.561	.428
Hostility	22.83	5.89	<0.001	<0.001	.280	<0.001	<0.001	<0.001	<0.001	1	.547
Depression	1.98	2.84	<0.001	<0.001	.005	<0.001	<0.001	<0.001	<0.001	<0.001	1

*Notes. N* = 544. PA = Psychological Abuse, PN = Psychological Neglect, PNS = Psychological Non-Support, ACE = Adverse Childhood Experience. Pearson correlations above the diagonal; *p*-values below the diagonal.

### Structural model of psychopathology

The hierarchical structural model of psychopathology shown in Fig. 1 provided, on balance, a good fit to the data (CFI = 0.985, TLI = 0.951, RMSEA = 0.100, SRMR = 0.027). This justified the use of the model for further stages of the analyses. In addition, the model met criteria for configural, metric and scalar measurement invariance by gender (Muthén & Muthén, 1998–2018; see Table 2). Subsequent analyses were thus run assuming scalar measurement invariance.

**Table 2:** . Configural, metric, and scalar gender invariance testing for the structural model of psychopathology.

Level of invariance by gender	CFI	TLI	RMSEA	SRMR	Satorra-Bentler *χ^2^*differenc test
Configural (invariant factor structure)	0.982	0.938	0.095	0.031	Configural vs. metric *χ^2^*(4) = 8.35, *p* = 0.079
Metric (invariant factor loadings)	0.978	0.956	0.095	0.044	Metric vs. scalar *χ^2^*(3) = 0.00, *p* = 1
Scalar (invariant intercepts and factor loadings)	0.978	0.937	0.114	0.044	Configural vs. scalar *χ^2^*(1) = 2.09, *p* = 0.148

*Notes. N* = 541. CFI = comparative fit index, TLI = Tucker-Lewis index, RMSEA = root mean square error of approximation, SRMR = Standardized Root Mean Square Residual.

### (Non-) equivalence of harm

When controlling for adverse childhood experiences, psychological abuse and psychological neglect had small to moderate positive and significant total effects on all symptom domains of psychopathology (from *b* = 0.265 to *b* = 0.386; see Table 3). Psychological non-support had small positive and significant total effects only on depression (*b* = 0.089) and anxiety (*b* = 0.278) (see Table 3). The results indicated that after controlling for adverse childhood experiences, all types of childhood psychological maltreatment had positive and significant associations with symptom domains of psychopathology. However, externalizing behaviors (i.e., anger, physical aggression, and hostility) did not have significant associations with psychological non-support.

**Table 3:** Standardized total effects, direct effects, and proportion of the variance accounted for by higher-order factors for different types of childhood psychological maltreatment with each domain of psychopathology when controlling ACE.

Domain of Psychopathology	Gender	Types of Childhood Psychological Maltreatment								
		Psychological Abuse			Psychological Neglect			Psychological Non-support		
		Total effect	Direct effect	Proportion unique	Total effect	Direct effect	Proportion unique	Total effect	Direct effect	Proportion unique
ACE	General	0.045	0.269*	–	0.041	0.271	–	0.061	0.383*	–
	Male	0.005	0.291*		−0.001	0.270*		0.007	0.282*	
	Female	0.081*	0.280*		0.073	0.291*		0.089	0.252*	
Depression	General	0.286*	0.025	8 %	0.284*	0.009	3 %	0.089 *	0.001	1 %
	Male	0.269*	0.325*		0.267*	0.206*		0.084*	0.116*	
	Female	0.298*	0.117		0.279*	0.038		0.084*	0.010	
Anxiety	General	0.289*	−0.074	0 %	0.386*	−0.020	0 %	0.278*	0.155	41 %
	Male	0.268*	0.170*		0.377*	0.171*		0.273*	0.317*	
	Female	0.305*	0.048*		0.394*	0.031*		0.271*	0.159*	
Anger	General	0.321*	−0.074	0 %	0.265*	−0.111	0 %	0.021	−0.015	–
	Male	0.285*	−0.122		0.252*	−0.118		0.023	−0.013	
	Female	0.323*	−0.043		0.261*	−0.099		0.023	−0.022	
Physical Aggression	General	0.354*	0.051	14 %	0.321*	0.055	17 %	0.005	0.019	–
	Male	0.308*	0.041		0.298*	0.056		−0.006	−0.004	
	Female	0.355*	0.040		0.313*	0.043		−0.006	0.001	
Hostility	General	0.297*	0.028	9 %	0.289*	0.067	23 %	−0.088	−0.095*	–
	Male	0.264*	0.105		0.270*	0.105		−0.083	0.105	
	Female	0.299*	0.001		0.286*	0.001		−0.084	0.001	
Internalizing	General	0.357*	−0.231	0 %	0.430*	0.014	3 %	0.266*	0.292*	109 %
	Male	0.425*	−0.252		0.496*	−0.037		0.267*	0.387*	
	Female	0.316*	−0.217		0.386*	0.082		0.250*	0.234*	
Externalizing	General	0.404*	0.160*	40 %	0.360*	−0.012	0 %	−0.005	−0.297*	–
	Male	0.458*	0.163*		0.501*	0.031		−0.076	−0.358*	
	Female	0.369*	0.156		0.266*	−0.078		0.024	−0.250*	
General Psychopathology	General	0.460*	–	–	0.479*	–	–	0.254*	–	–
	Male	0.505*	–		0.573*	–		0.250*	–	
	Female	0.425*	–		0.413*	–		0.247*	–	

Notes.

⁎ = <0.05.

### Outcome specificity

When controlling for adverse childhood experiences large proportions of the total effect for psychological abuse (86 % - 100 %), and psychological neglect (77 % - 100 %) were consistently accounted for by the higher-order factors. Psychological non-support showed that a 59 % proportion of anxiety and 99 % proportion of depression of the total effects was consistently accounted for by the higher-order factors. All the direct effects – representing the part of the total effect unique to each symptom domain were non-significant (see Table 3).

Moving to the next level of the higher-order model, there were positive and significant total effects of different types of psychological maltreatment on internalizing and externalizing behaviors (from *b* = 0.357 to *b* = 0.430). A large proportion (60 %-100 %) of each effect was consistently accounted by for the general psychopathology factor. All the other direct effects were non-significant or significant but negative. However, the direct effects of psychological abuse and of psychological non-support on externalizing behavior was significant (see Table 3).

Finally, moving to the next level of the higher-order model, the effects of different types of psychological maltreatments on general psychopathology were all positive and significant but weaker (*b_PA_* = 0.460, *b_PN_* = 0.479, *b_PS_* = 0.254; see Table 3).

**Fig. 2:**
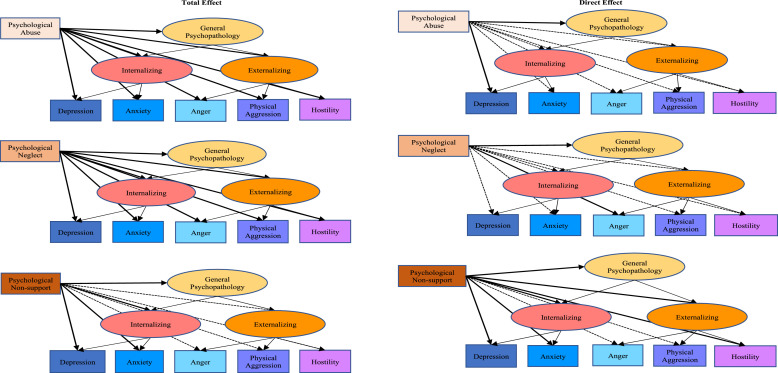
Visual summary of the total versus direct effects (parameters specified in Table 3) for each type of childhood psychological maltreatment with each domain of psychopathology when controlling adverse childhood experiences. Solid lines represent significant effects, and dashed lines denote non-significant effects at *p* < 0.01. Note that the direct effect for general psychopathology is equal to the total effect, as there are no higher-order variables controlled for in this association.

Taken together, these results suggested that the associations between different types of psychological maltreatment and psychopathology are not unique to specific symptom domains but associated with all symptom domains captured by an association with internalizing, externalizing and general psychopathology (see Fig. 2 for visual summary of the parameters presented in Table 3 and Table 3).

### Gender invariance

Gender was examined as a moderator in all analyses using a multi-group framework that compared models with regression paths constrained versus unconstrained across males and females. All models were invariant by gender, with all ΔCFI < 0.01 and Satorra-Bentler chi-squared difference tests non-significant except the association between psychological non-support and hostility, for which gender invariance did not hold.

## Discussion

Experiences of psychological maltreatment are common during childhood and have been associated with a broad variety of psychopathology outcomes in adulthood. The current study examined the hypotheses that (1) psychological abuse and psychological neglect would be more strongly associated with psychopathology than psychological non-support *((non-) equivalent harm?)*; (2) different forms of psychological maltreatment would have broadband associations with specific domains of psychopathology (i.e., depression, anxiety, anger, physical aggression, and hostility) *(outcome specificity)*; and (3) the strength of these associations would not vary by gender *(gender invariance?)*. As hypothesised, the results suggested that psychological abuse and psychological neglect were more strongly associated with psychopathology than psychological non-support. In addition, the results indicated that psychological abuse and psychological neglect had broadband associations across all common domains of psychopathology, including at all levels of a hierarchical model of psychopathology after adjusting for adverse childhood experiences. Psychological non-support, on the other hand, was only associated with general psychopathology, internalizing problems, and anxiety after controlling for adverse childhood experiences, supporting the non-equivalence assumption. Finally, the results suggested that these associations were invariant by gender, with the exception of the associations between psychological non-support and hostility – males showed negative but significant associations between psychological non-support and hostility, while females did not show any significant association.

## Implications

Equivalence of harm was indicated by the findings that childhood experiences of psychological abuse and psychological neglect had similar, small to moderate relationships with all symptom domains of psychopathology examined. The comparisons of harmfulness between childhood psychological abuse and psychological neglect have been rare in previous studies. Previous studies have suggested that psychological neglect has weaker associations with internalizing problems than psychological abuse (Vahl et al., 2016). In contrast, the current study found psychological neglect was more strongly associated with internalizing problems than psychological abuse.

Psychological neglect is a prevalent form of abuse (Taillieu et al., 2016); however, it receives less attention than others. The relative lack of prevention and intervention efforts related to psychological neglect may be because acts of omission (psychological neglect) are more difficult to identify than acts of commission (psychological abuse) (Chamberland et al., 2011). Recognizing and responding to psychological maltreatment in all its forms is important and a priority for public health. Given the potential harmfulness of psychological maltreatment during childhood, parents or other caregivers need to be aware of the consequences of psychological maltreatment towards children, and its possible long-term on mental health. For example, parenting programs for preventing psychological maltreatment are promising approaches for preventing and reducing the potential negative impacts on mental health (e.g., Cluver et al., 2018). However, these parenting programs do not tend to measure psychological maltreatment outcomes other than emotional abuse, which and it will be important to expand the outcome measures for these studies to include the broader set of potentially harmful parenting practices. More generally, an effective parenting program may need to integrate both efforts to reduce non-physical forms of aggression by parents or caregivers (i.e., promoting positive parenting, supportive and trust bonding with child) and reduce different forms of child psychological maltreatment.

In the current study, all levels of the hierarchical model of psychopathology were associated with childhood psychological abuse and psychological neglect, with the strongest associations with general psychopathology, followed by internalizing and externalizing factors. There was no evidence for outcome specificity, as most of the domain-specific relationships were accounted for by the associations between psychological maltreatment and general psychopathology. There is lack of literature exploring whether psychological maltreatment is a trans-diagnostic risk factor in psychopathology. In one of only a small number of studies to address this issue, Keyes et al. (2012) found that different forms of child maltreatment predicted internalizing and externalizing problems rather than symptoms of specific psychiatric disorders. Our results were consistent with this and thus are in line with the limited evidence base on this thus far available.

Several studies examining childhood adversity more broadly (Conway et al., 2018; Schaefer et al., 2018) draw similar conclusions – suggesting that childhood adversities such as domestic violence, bullying by peers, sexual abuse, physical abuse, emotional abuse, and early childhood stress are associated with broad factors such as internalizing, externalizing, or general psychopathology. The current study suggested the generality of the risks conferred by childhood psychological maltreatment as well, indicating that developing broadband preventive intervention and trans-diagnostic treatment may be the most efficacious approach to mitigating the mental health harms of childhood psychological maltreatment.

Some previous research has suggested gender differences in the association between childhood psychological maltreatment (Vahl et al., 2016); however, other research has reported evidence conflicting with this. For instance, Li, Zhao and Yu (2022) found no gender differences in the relations between childhood emotional abuse and depression. In line with the previous literature, the current study found only minimal evidence for gender as a moderator of the relations between childhood psychological maltreatment and mental health outcomes, limited to the relations between hostility and psychological non-support which were only present for males. This suggests that the negative impacts of psychological maltreatment are largely universal across males and females, consistent with the evidence on other forms of child abuse (Vachon et al., 2015).

## Strengths and limitations

There are some limitations that should be considered. The primary limitations are regarding measurement. First, the reliance on retrospective self-report for recalling childhood psychological maltreatment experiences may be subjective to biases. Several empirical studies have investigated the biases of adulthood recalled childhood adversity experiences, finding inconsistencies between prospective and retrospective reporting (e.g., Colman et al., 2016). However, Hardt and Rutter (2004) concluded that although there were false negative experiences and substantial measurement error on recalling childhood adversity, the errors or biases were not strong enough to invalidate retrospective measurement. Nevertheless, replicating the present findings using alternative approaches, including prospective data collection and multi-informant perspective will be important.

Further research should also include the measurement of substance abuse. Substance abuse as a common expression of externalizing behaviors and has been well-investigated as an outcome of childhood psychological maltreatment. Extant research has indicated that participants with childhood psychological abuse experiences are more likely to be engaged in different kinds of substance abuse such as alcohol (Crouch et al., 2012), cannabis (Aas et al., 2014), nicotine (Elliott et al., 2014), or heroin Afifi et al. (2012). A systematic review (Xiao et al., 2022a) suggested that the effect size for the associations between substance abuse and childhood psychological abuse were moderate to strong (from 0.30 to 0.85), while the effect size for childhood psychological neglect were small to strong (from 0.15 to 0.66). Moreover, in the current study, we did not ask participants if they were devoid of medications or exposure to other interventions; therefore, we were not able to investigate whether the medications or interventions may impact their symptoms.

In the current study, we recruited from the general population from different regions in China, but we did not recruit from clinical populations. Previous literature has suggested that clinical populations may have suffered more childhood psychological maltreatment than general populations. This includes higher abuse prevalence in clinical population such as those with major depression (de Mattos Souza et al., 2016), personality disorders (Zhang et al., 2013), eating disorders (Amianto et al., 2018), or PTSD (Evren et al., 2016). A systematic review conducted by Xiao et al. (2022a) showed that the small to moderate effect size of the associations between childhood psychological maltreatment and having a clinical psychopathology diagnosis. It would, therefore, be valuable to replicate the current study in high-risk population (i.e., high risk of exposure to childhood psychological maltreatment) or clinical populations to examine whether the findings reported here generalize to these populations.

## Conclusion

Taken together, the results indicated that experiences of childhood psychological maltreatment, may pose a broadband risk for any and all forms of psychopathology. With no physical scars, no standard definition, and challenges in legislating against it, it can be difficult to detect, recognize, prevent, and intervene on this risk factor. Given its broad and lasting negative impact into adulthood, research and policy should place greater emphasis on psychological maltreatment, which occurs with high frequency; however, children receive less formal protection against it than other types of child maltreatment.

## Declaration of Competing Interest

The author(s) declared no potential conflicts of interest with respect to the research, authorship, and/or publication of this article.

## Appendix

Image, application 1
