# Supplementary material for: Relations between childhood psychological maltreatment and mental health dimensions within a higher-order model
Source: Int J Clin Health Psychol. 2023 Oct 7;24(1):100416. doi: 10.1016/j.ijchp.2023.100416 (PMC10563048; doi:10.1016/j.ijchp.2023.100416)
Supplement: Supplementary file 1 [file mmc1.docx]

| Table S1. Demographic Characteristics | | | |
| --- | --- | --- | --- |
|  |  | N | % |
| Female |  | 347 | 63.90% |
| Male |  | 197 | 36.00% |
| Non-binary |  | 3 | 0.5% |
| Age |  |  |  |
| 18-20 |  | 114 | 20.80% |
| 21-30 |  | 328 | 60.00% |
| 31-40 |  | 90 | 16.50% |
| 41-60 |  | 14 | 2.60% |
| Over 61 |  | 1 | 0.20% |
| Primary Caregivers |  |  |  |
| Mother |  | 346 | 63.3% |
| Father |  | 107 | 19.6% |
| Stepmother |  | 4 | 0.70% |
| Stepfather |  | 1 | 0.20% |
| Grandparents |  | 74 | 13.50% |
| Nanny |  | 6 | 1.10% |
| Others |  | 9 | 1.60% |
| *Notes.* N = 544. Others refer to other relatives such as uncle or aunt. For Male, 20.8% were between 18-20, 59.9% were between 21-30, 17.3% were between 31-40, and 2% were between 41-60. For Female, 20.6% were between 18-20, 60.2% were between 21-30, 16.3% were between 31-30, and 2.9% were between 41-60. | | | |

| Table S2. ANOVA of ACE on different age group | | | | | | |
| --- | --- | --- | --- | --- | --- | --- |
|  |  | Sum of Squares | df | Mean Square | F | p |
| ACE | Between Groups | 1.70 | 3 | .566 | .380 | .768 |
|  | Within Groups | 760.86 | 510 | 1.50 |  |  |
|  | Total | 762.56 | 513 |  |  |  |
